# Functional cure with single agent olutasidenib in relapsed IDH1/NPM1 co-mutated AML

**DOI:** 10.1038/s41698-025-01013-5

**Published:** 2025-07-01

**Authors:** Justin Watts, Tiffany Nong, Katarina Micin, Deborah Soong, Ellen Madarang, Maurizio Affer, Shefali Mehra, Jessica Alvarez Lesmes, Jennifer Chapman, Yi Zhou, Amber Thomassen, Terrence Bradley, Tulasigeri Totiger, Ronan Swords, Justin Taylor

**Affiliations:** 1 University of Miami Sylvester Comprehensive Cancer Center, Division of Hematology, Miami, FL USA; 2 University of Miami Sylvester Comprehensive Cancer Center, Department of Pharmacy, Miami, FL USA; 3 University of Miami Miller School of Medicine, Department of Pathology, Miami, FL USA; 4 Oregon Health & Science University, Knight Cancer Institute, Division of Hematology/Medical Oncology, Portland, OE USA

**Keywords:** Cancer, Molecular medicine, Oncology

## Abstract

Olutasidenib is a potent, selective, oral, small-molecule inhibitor of mutant isocitrate dehydrogenase 1 (IDH1) that was recently approved by the US FDA for adult patients with relapsed or refractory acute myeloid leukemia (AML) harboring mutant IDH1. In the pivotal Phase II trial of olutasidenib, the median duration of complete response (CR) was 28.1 months. Here we report the first patient in the world to receive olutasidenib, for relapsed NPM1 and IDH1 co-mutated AML, who remains in continuous CR for over 7 years on olutasidenib monotherapy. We detail the clinical course as well as the pathologic and genomic evolution of the disease. Furthermore, using a novel single cell measurable residual disease assay and digital PCR and qPCR for the detection of IDH1 and NPM1 mutations, we found no evidence of residual detectable leukemia. To our knowledge, this is the first report of an AML patient functionally cured by IDH1 inhibitor monotherapy.

## Introduction

Acute myeloid leukemia (AML) is a hematopoietic stem and progenitor cell (HSPC) malignancy characterized by clonal and irreversible expansion of myeloid blasts through aberrant differentiation and uncontrolled proliferation^1,2^. It typically affects the elderly, with a median age of 69 years at diagnosis and a median age of death of 73 years^3^. Disease cytogenetics predicts response to induction chemotherapy, relapse risk, and overall survival (OS)^4–7^. However, incorporation of somatic gene mutations has improved the accuracy of AML risk stratification^1,8^. Discovery of these mutations allowed for pharmacological advancement in small molecular inhibitors such as those targeting mutated isocitrate dehydrogenase 1/2 (IDH1/2) and changed the treatment landscape of relapsed/refractory AML outcomes.

Mutations in IDH1 and IDH2 occur in approximately 6-10% and 9-13% of patients with AML, respectively^2^. IDH1 and IDH2 are metabolic enzymes that catalyze the oxidative decarboxylation of isocitrate to alpha-ketoglutarate (α-KG). Gain-of-function mutations in IDH1/2 instead reduce isocitrate to R-2-hydroxyglutarate (2HG), which functions as an oncometabolite promoting differentiation block and cytokine-independent cell proliferation by reshaping the epigenomic landscape^9–12^. Mutations occur at conserved arginine residues in the activating domain of the enzymes: specifically, R132 of IDH1, and R140 or R172 of IDH2^13,14^. The impact of mutant IDH on AML prognosis is typically considered favorable, though it can be associated with poor prognosis in other malignancies^15,16^. In addition, IDH1 mutant patients may have inferior outcomes compared to mutant IDH2 patients when treated with either standard induction chemotherapy or venetoclax and azacitidine^10,16–18^.

Relapsed/refractory (R/R) AML is associated with poor clinical outcomes, with a 3-year overall survival (OS) rate of less than 10%^19^. Most patients who relapse will do so within three years of initial diagnosis and fewer than 3% will experience a late relapse five years or more after achieving complete remission (CR)^20,21^. Ivosidenib was the first FDA-approved targeted agent against mutant IDH1, and robustly suppresses the synthesis of 2HG^22,23^. In a Phase I expansion study investigating the use of ivosidenib monotherapy in 125 R/R IDH1-mutated AML patients (NCT02074839), the CR rate was 21.6% and the median duration of complete remission was 9.3 months^22^. Mechanisms for ivosidenib resistance have recently been reported, and include signaling pathway gene mutations (e.g., *NRAS, KRAS, PTPN11, FLT3*-ITD*)*, which are associated with primary treatment resistance, and the emergence of such mutations was also associated with secondary resistance to ivosidenib at relapse/progression^24^. The emergence of IDH1 second-site mutations and isoform switching to IDH2 have also been reported as potential resistance mechanisms^25^.

Olutasidenib is a highly potent, selective, oral small-molecule inhibitor of IDH1 that was FDA-approved for R/R IDH1-mutated AML in December 2022. In the pivotal Phase II study (NCT02719574) of 153 IDH1 inhibitor-naïve R/R AML patients with an IDH1 R132 mutation, the rate of CR was 32% and the median duration of CR was 28.1 months^26^. This median duration of remission is remarkably durable for a single agent targeted therapy in R/R AML, surpassing any other agent in its class and any reported FLT3 inhibitor. Recent 5-year follow-up data from the pivotal Phase II trial further support that there may be other very long-term responders to olutasidenib, although this sub-group has yet to be fully analyzed (e.g., median follow-up in responders, rates of allogeneic stem cell transplantation [allogeneic-SCT] in long-responders)^27^.

In this case report, we describe a 66-year-old female with AML who initially achieved complete remission following induction chemotherapy with the “7 + 3” regimen of cytarabine and daunorubicin, followed by four cycles of high-dose cytarabine consolidation. Unfortunately, she later experienced a relapse, now harboring an IDH1 R132C mutation and concomitant NPM1 mutation. She was the first patient enrolled in the original first-in-human Phase I clinical trial of single agent olutasidenib, achieving a rapid hematologic response after two cycles and complete response after three cycles, with clinical next-generation sequencing (NGS) showing resolution of all pathogenic mutations. She has remained on olutasidenib in continuous CR for over 7 years. Although other patients from the Phase I and pivotal Phase II trials of olutasidenib have also had prolonged remission duration (as evidenced by the median duration of CR), it is unknown if these remissions translate to cure without allogeneic-SCT. This case presents evidence that long-term remission and functional cure can be achieved with single agent olutasidenib therapy.

## Results

### AML clinical course and pathologic findings at initial diagnosis

A 66-year-old female with a history of Hemoglobin C and S trait and irritable bowel syndrome initially presented with persistent pharyngitis for over two weeks, with complete blood count showing white blood count (WBC): 1.0 × 109/L, hemoglobin (Hgb): 9.3 g/dL, hematocrit (Hct): 26.9%, platelets (Plt): 62 × 109/L. She was diagnosed with acute myeloid leukemia 11 years prior with a diagnostic bone marrow biopsy showing hypercellular marrow replaced by sheets of blast (95% myeloid blasts) with flow cytometry showing immunophenotype positivity for CD45, MPO, CD117 (dim), CD33, CD13 (DIM), and CD39 (DIM), and negative for CD34 and HLADR. PCR analysis revealed a positive NPM1 mutation, and IDH1 testing was not performed at that time. A fluorescence in situ hybridization (FISH) panel showed partial deletions of the long arms of chromosomes 5, 7, and 20. Chromosomal analysis showed an abnormal female karyotype 46,XX,r(7)(p22q32) [2])/46, XX [18], characterized by a ring chromosome 7. The patient received induction chemotherapy with standard doses of cytarabine and daunorubicin and achieved first CR (CR1). Bone marrow biopsy performed post-induction showed normal appearing blasts that comprised 1% of non-erythroid cells by flow, with karyotype 46,XX [20], and NPM1 mutation not tested. She subsequently received 4 cycles of high-dose cytarabine consolidation and tolerated it without complications. She did not proceed to allogeneic-SCT due to a lack of donor options.

### AML clinical course, disease characteristics and pathomolecular findings at relapse

**Fig. 1:**
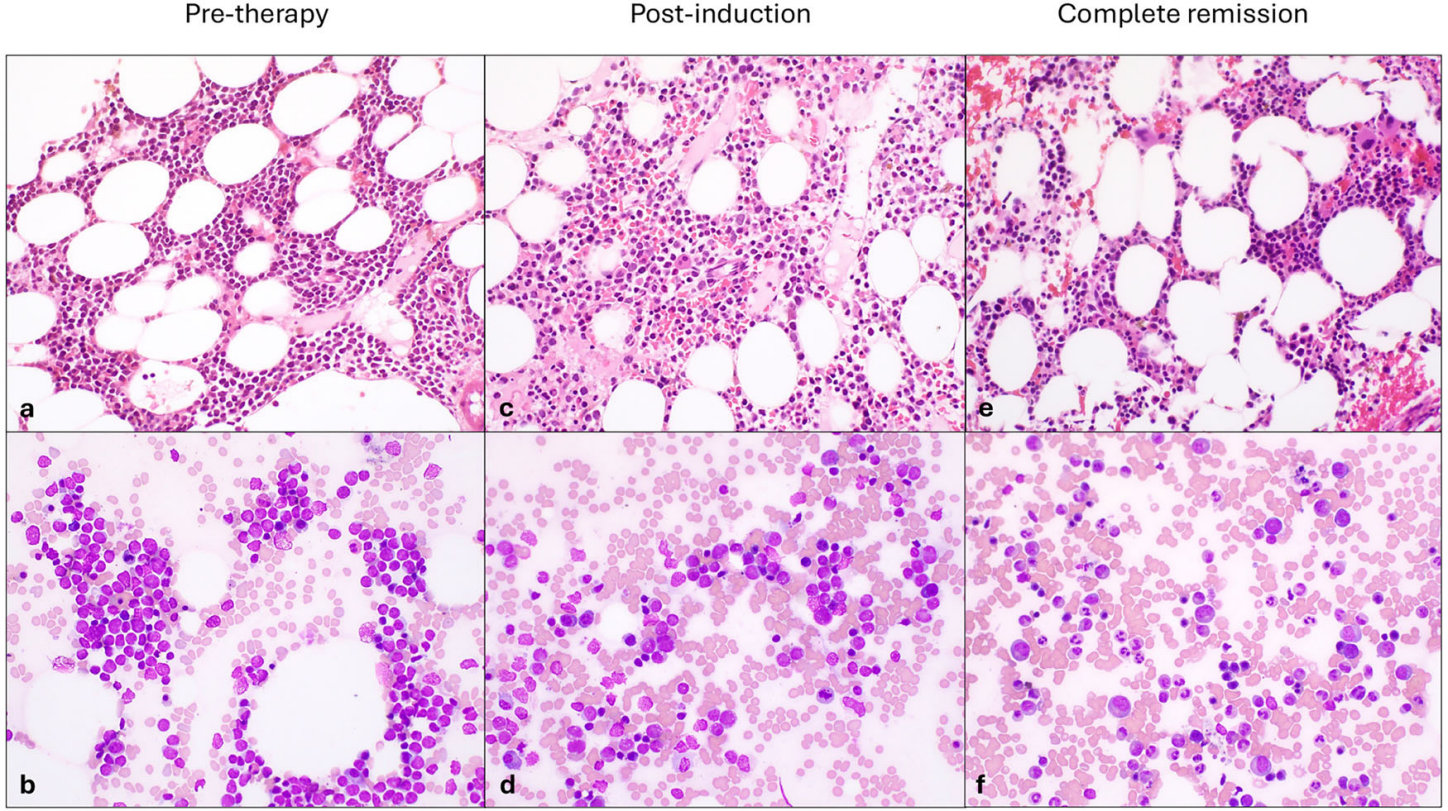
Bone marrow biopsy before, during and after treatment with olutasidenib. Representative sections of bone marrow core biopsy and bone marrow aspirates. **a**, **b** Numerous blasts are present in the baseline bone marrow sample. **c**, **d** Post-induction bone marrow with persistent but decreased blasts with the presence of myeloid differentiation. **e**, **f** Post-therapy bone marrow showing remission with maturing trilineage hematopoiesis.

Approximately 4 years later, the patient developed new symptoms, including persistent sore throat and notable voice change. The complete blood count showed WBC: 1.8 ×109/L, Hgb: 9.2 g/dL, Plt: 158, ANC: 0.56 ×109/L. She underwent repeat bone marrow biopsy that showed 70% myeloid blasts, positive for dim CD45, CD117, CD33, CD13, and CD14, and negative for TdT, CD34, MLADR, CD19, and CD3 consistent with relapsed AML (Fig. 1a, b). Cytogenetics testing showed 46,XX,add(1)(q32),t(2;5)(q11.2;q31),r(7)(p22q32),del(16)(p11.2) [7]/46,XX [13]. NGS revealed mutations in NPM1 (c.860_863dupTCTG; p. W299Cfs*12, 19% variant allele frequency [VAF]), and IDH1 (c.394 C > T, p.R132C, 20% VAF).

### Therapeutic response to olutasidenib in AML with IDH1 R132C mutation

Given the new IDH1 R132C mutation, the patient was enrolled in the Phase I first-in-human investigational trial of olutasidenib, an allosteric inhibitor of IDH1^mut^. She was assigned treatment with olutasidenib at the 150 mg daily level in continuous 28-day cycles, which is half of the eventually approved therapeutic dose. After the first 28-day cycle, the patient developed neutropenic fever, anemia and thrombocytopenia requiring both packed red blood cell transfusions and platelet transfusions. Bone marrow biopsy after cycle 1 showed a reduction in blasts to 24%, abundant myeloid differentiation (Fig. 1c, d), and resolution of the ring 7 chromosome but remaining imbalanced t(2;5). Her blood counts returned to normal during cycle 2, and after cycle 3, bone marrow biopsy showed a complete response with maturing trilineage hematopoiesis (Fig. 1e, f) and clearance of all cytogenetic abnormalities with a normal female karyotype 46,XX [20]. This was now her second CR (CR2). The patient continued to have excellent functional status with ECOG 0 throughout treatment, and did not have differentiation syndrome. Bone marrow biopsy after 12 cycles of treatment showed no morphologic evidence of AML with 0.2% normal-appearing blasts on flow and normal cellularity. Karyotype was 46,XX [20], and standard NGS did not show any pathogenic alterations, including the absence of NPM1 and IDH1 mutations.

The patient continued to be in complete response on olutasidenib 150 mg daily, with bone marrow biopsies after cycles 24, 32, and 38 with normal karyotype 46,XX [20] and pathogenic alterations not detected on the NGS panel. The most recently performed bone marrow biopsy after cycle 84 continued to show normocellular bone marrow ( ~ 30%) with 0% blasts, normal karyotype 46,XX [20], and no pathogenic alterations on NGS. After olutasidenib was approved by the FDA on December 1^st^, 2022, for adult patients with R/R AML with susceptible IDH1 mutation, the patient was transitioned to commercial supply. She continues to tolerate olutasidenib 150 mg daily for a total duration of olutasidenib treatment of 87 months ( > 7 years from first relapse) and over 11 years from the initial AML diagnosis. The patient continues olutasidenib with complete blood count measured every 8 weeks and clinical examination every 6 months.

### Deep MRD negative remission after seven years of olutasidenib therapy

**Fig. 2:**
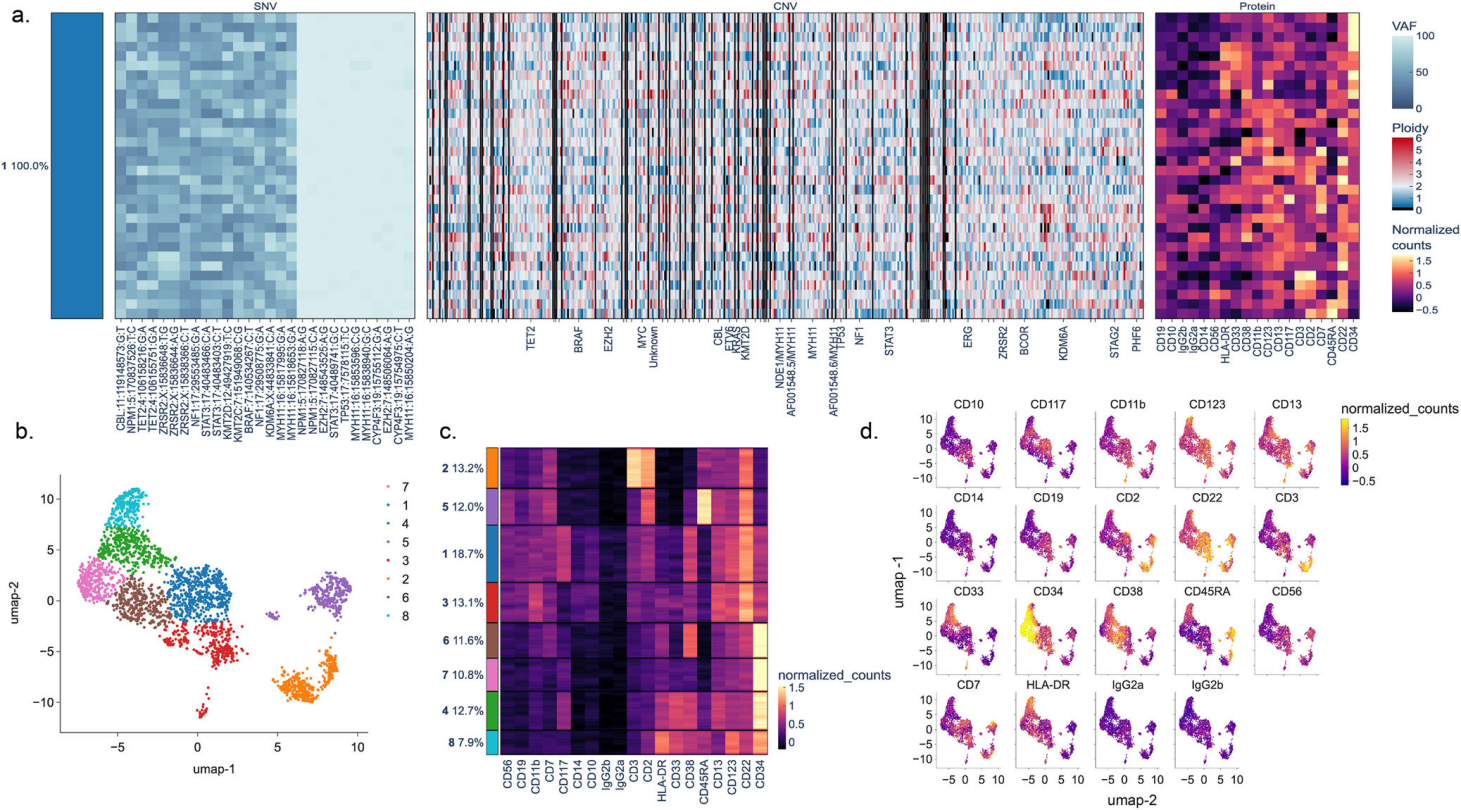
Comprehensive analysis of MRD negativity using single cell MRD sequencing in relapsed AML patient post olutasidenib. **a** Multimodal heatmap illustrating the detected single nucleotide variants (SNV), copy number variant (CNV) ploidy estimation, and antibody-oligonucleotide conjugated (AOC) normalized counts distribution in the patient post- olutasidenib. Numerous single nucleotide polymorphisms and copy number variants were present, but the previously detected NPM1 and IDH1 AML-associated mutations were notably absent. **b** Louvain-based clustering identified 8 distinct protein clusters. **c**, **d** Noise Corrected and Scaled (NSP)-normalization AOC counts distribution does not indicate any leukemic or AML-associated surface protein expression. The absence of any AML-associated mutant or phenotypic clones at single cell resolution, further verifies the patient’s MRD negativity post-olutasidenib.

To assess the depth of response and for any remaining measurable residual disease (MRD), we performed several studies on the most recent bone marrow biopsy sample from after cycle 84. High-sensitivity quantitative NPM1 PCR (qPCR, Neogenomics) did not detect the mutation. To confirm deep MRD remission, we also performed novel, highly sensitive assays including single cell MRD (scMRD) and digital PCR (dPCR) for IDH1 and NPM1. The scMRD assay uses a multiomics approach that incorporates both genotypic and immunophenotypic analyses to distinguish between clonal hematopoiesis and residual disease. This assay includes a CD34 + /CD117+ enrichment and in this case captured 3171 enriched single cells and detected no AML-associated mutant cells or phenotypic clones (Fig. 2a–d). Of the single nucleotide variants (SNVs) revealed by scMRD, both the IDH1 and NPM1 variants detected at relapse were absent. The detected SNVs included: TET2:chr4:106155751:G/A, TET2:chr4:106158216:G/A, EZH2:chr7:148504716:AG/A, EZH2:chr7:148506064:A/G, EZH2:chr7:148543525:A/G, CBL:chr11:119148573:G/T, MYH11:chr16:15817995:G/A, MYH11:chr16:15838940:G/C, MYH11:chr16:15850204:A/G, and MYH11:chr16:15853596:C/G, which are not pathological variants associated with AML and are not indicative of residual disease.

**Fig. 3:**
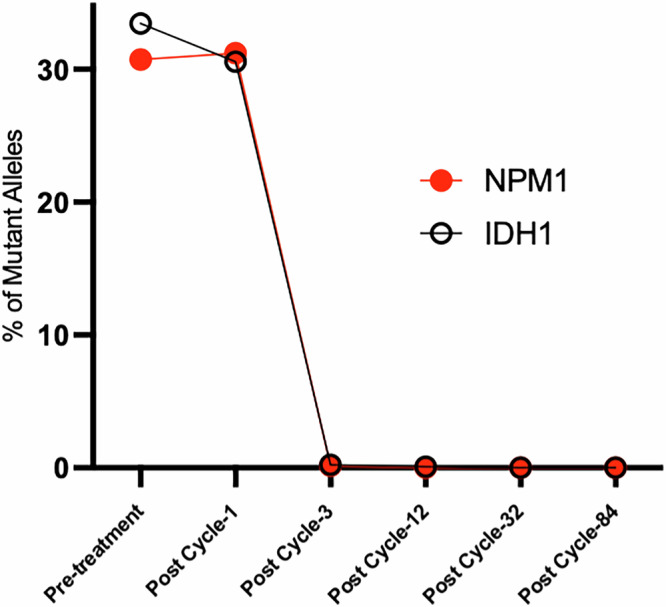
Assessing the presence of *NPM1* and *IDH1* mutant alleles using digital PCR in an AML patient from relapse to long-term remission. Digital PCR was performed for *NPM1* and *IDH1* mutations at the indicated timepoints.

To confirm the absence of any AML-associated NPM1 and IDH1 variants in the patient, we employed dPCR for precise and sensitive detection, ensuring that no trace of these variants was present on serial bone marrow samples since the achievement of CR2. The assay is based on a set of two probes and primers specific for each gene, with one probe detecting the wild type and the second one the mutant form of IDH1 and NPM1, respectively. The results showed that at relapse and after the first cycle of olutasidenib, both mutations were present at approximately 30%; however, for the next four time points available after CR2, the presence of the mutant forms of the two genes was not detectable by dPCR spanning a period of 7 years (Fig. 3).

## Discussion

Due to the heterogeneity of AML, precision medicine targeting specific driver mutations has gained traction, leading to the approval of mutant FLT3, IDH1, and IDH2 inhibitors, as well as the emerging promise of menin inhibitors^28^. The treatment of AML typically requires multiple lines of chemotherapy with the incorporation of novel targeted agents until disease progression^29^. Olutasidenib is an allosteric inhibitor of IDH1-R132 variants that binds non-competitively to the hydrophobic pocket of the IDH1 heterodimer interface^30,31^. This case report presents a 66-year-old female patient with NPM1-mutated AML who achieved CR1 but relapsed at age 71 with NPM1 and IDH1 R132C co-mutated AML. The patient achieved CR2 after 3 months of treatment initiation with single agent olutasidenib, a highly selective mutant IDH1 inhibitor, and has remained in continuous CR2 for over 87 months, an exceptionally durable duration of response. This response duration far surpasses the already impressive median CR duration of 28.1 months (95% CI, 13.8-NE) reported in the pivotal Phase II trial, which led to the approval of olutasidenib (NCT02719574)^26^. To our knowledge, this is the first report of a patient with such an exceptionally durable response to any single agent targeted therapy for R/R AML.

Accurately measuring residual disease is a challenge that continues to be incrementally improved in AML, where multiple driver mutations may interact in its pathogenesis^32,33^. This patient’s achievement and maintenance of deep MRD-negative remission was confirmed by highly sensitive assays such as scMRD, digital PCR (dPCR) and qPCR, which make it particularly noteworthy. Here, we used a scMRD assay that sequenced 3171 hematopoietic stem and progenitor cells (HSPCs). This scMRD assay is highly sensitive for detecting residual mutations, clonality, and aberrant phenotypes, thus confirming the true absence of measurable residual disease^34^. This is a novel MRD assay that we have implemented on prospective sample collection and for real-time clinical use in addition to current standard of care methods. This deep molecular response underscores the exceptional efficacy of olutasidenib in this case.

After the patient’s first cycle of olutasidenib, common disease-related adverse events observed in the Phase II study occurred, including febrile neutropenia, anemia, and thrombocytopenia, which required both packed red blood cell and platelet transfusions^26^. The patient did not have differentiation syndrome or olutasidenib-specific toxicity. The topic of treatment-free remission has not yet been established in AML, particularly with IDH inhibitors, but considering the duration of this patient’s remission and deep molecular clearance demonstrated by MRD PCR-techniques and scMRD (NGS-based), we question whether it may be a possibility. Given this patient’s exceptional response and the long duration of CR and CR/CRh (complete remission with partial hematologic recovery) observed in large cohorts of R/R AML patients treated with single agent olutasidenib, the future holds promise for the development novel regimens that may induce treatment-free remission in R/R and even frontline IDH1 mutated AML settings, by incorporating olutasidenib into combination regimens. Although not investigated in a head-to-head trial, olutasidenib demonstrated more CRs and significantly longer CR and CR/CRh durations compared to ivosidenib, along with superior overall survival in responding patients^26^. Clinical trials are underway investigating hypomethylating agents and the BCL-2 inhibitor venetoclax in combination with olutasidenib (NCT06445959).

This patient was the first patient in the world treated on the initial Phase I first-in-human clinical trial of olutasidenib at the starting dose level of 150 mg daily (the on-label starting dose is 150 mg BID) for R/R IDH1 mutated AML. The question arises as to why this patient had such an exceptional response. Critically, and despite a high blast percentage of 70% at relapse, she had only two driver mutations (NPM1 W299Cfs*12 and IDH1 R132C) with similar VAFs of 19% and 20%, respectively, without a co-occurring FLT3 or RAS pathway mutation. Her molecular presentation highlights at least three key take-aways: 1.) the total mutational burden, 2.) the absence of signaling pathway mutations, and 3.) the clonal hierarchy of the IDH1 mutation may both individually and collectively influence the duration of response to single agent IDH1 inhibition^24,25,35^. Furthermore, the clonal positioning of the patient’s IDH1 mutation may have been particularly favorable, as it appears to be in every cell with a mutant NPM1 gene based on the equivalent VAFs, without the presence of a dominant clonal hematopoiesis (CH) clone or a detectable sub-clonal event to cause outgrowth of resistant clones. The ring chromosome 7 she had at diagnosis and relapse is rare but has been reported to be associated with poor prognosis in AML, but it resolved after one cycle of olutasidenib; and after three cycles she had a normal karyotype, suggesting that it was more of a passenger and not the disease driving abnormality. To date, NPM1 mutations have not been associated with a favorable response to IDH1 inhibitors. In fact, the opposite appears to be true, but this relationship is difficult to determine due to the common presence of co-occurring FLT3-ITD mutations, and more data are needed^36^. In the pivotal Phase II trial of olutasidenib, the R132C variant was the most common IDH1 mutant isoform at 61%, and 21% of all patients had a co-occurring NPM1 mutation, similar to our patient. IDH1 R132C has been associated with a more favorable clinical outcome than the R132H subtype, including improved OS after allogeneic-SCT^37^ and higher response rates with olutasidenib^26^. Our patient also had a “type A” NPM1 mutation, whereas non-ABD NPM1 mutations have been associated with inferior outcomes to chemotherapy^38^. It is also notable that our patient experienced a late first relapse ~4 years after prior intensive chemotherapy, which perhaps contributed to her lack of clonal complexity and the ensuing sensitivity to olutasidenib monotherapy. Lastly, this patient’s exceptional response may be attributed to distinct biochemical properties of olutasidenib that differentiate it from other IDH1 inhibitors, such as its small molecular weight, 2:1 binding stoichiometry, pharmacokinetic differences, strong selectivity for mutant IDH1 dimers over wild-type, and potent inhibition of IDH1 second-cite mutations^30^. The final 5-year follow-up data from the pivotal Phase II olutasidenib cohort suggested the presence of a significant subset of additional very long responders ( ~ 45% of CR/CRh patients, Kaplan-Meier estimate), which deserves deeper analysis^27^.

In conclusion, this case report illustrates the potential of olutasidenib to induce markedly durable long-term remissions in patients with IDH1 mutant AML. Molecular testing is necessary and now part of the standard of care for treating AML, and the exceptional response observed in this patient underscores the importance of targeted therapies and the incorporation of highly sensitive MRD assays. Moreover, single cell MRD resolution may help differentiate persistent clonal hematopoiesis from residual leukemic clones. The limitation of this study is the single case nature. As our understanding of the molecular and therapeutic landscape of AML continues to evolve, striking cases like this one remind us of the hope and promise of clinical trials and provide insight to inform clinical decision making and direct future research efforts, such as exploring treatment-free remission in AML patients who respond to targeted therapies and have no measurable residual disease by extra-sensitive methods.

## Methods

### Consent to publish

The patient received treatment under regular clinical care but retrospectively provided written informed consent to be included in this study. Written informed consent for publication of their clinical details was obtained from the patient. A copy of the consent form is available for review by the Editor of this journal.

### Single cell MRD library preparation and sequencing

Patient sample processing for single cell sequencing was performed using the Mission Bio TapestriⓇ platform. The bone marrow aspirate was enriched for CD34+ and CD117+ cells using MicroBeads based Miltenyi enrichment kit (Cat# 130-046-702 and 130-091-332, respectively) and then multiplexed and prepared for genomic and protein library generation using Tapestri v3 chemistry according to the manufacturer’s instructions. The samples were then sequenced on an Illumina® NovaSeq X Plus.

### scDNA and surface protein computational analysis

Multiplexed DNA and protein FASTQ files were processed using the Tapestri MRD-AML DNA + Protein v1.0.2 pipeline. This pipeline performs adaptor sequence trimming, aligns all sequencing reads to the hg19 reference genome, assigns each sequencing read to a cell barcode and performs DNA variant calling. Using patient sample-specific germline variants, demultiplexing was carried out and individual sample data was provided in an h5 file format. The patient-specific h5 file was then loaded into the Mission Bio DNA + Protein analysis Jupyter notebook, using Mosaic version 3.4. DNA variant filtering was performed to exclude any variants that did not meet assigned quality control cutoffs from downstream analysis. Filtering cutoffs included minimum read depth (min_dp) = 10, minimum genotype quality (min_gq) = 30, reference variant allele frequency (vaf_ref) = 5, variant allele frequency for homozygous variants (vaf_hom) = 95, variant allele frequency for heterozygous variants (vaf_het) = 30, minimum percentage of cells the variant is present in (min_prct_cells) = 50, minimum percentage of cells the mutant allele is present in (min_mut_prct_cells) = 1, and number of filtering iterations = 10. Additionally, all final DNA variants used in downstream analysis had an allelic dropout score (ADO) ≤ 1 and were present in ≥ 1% of the sample cell population.

### Digital polymerase chain reaction

Digital polymerase chain reaction was performed testing genomic DNA from specimens carrying a R132C IDH1 mutation (CGT > TGT) and an NPM1one where there is a duplication of the tetranucleotide TCTG between position 860 and 861. Briefly: genomic DNA was added to a master mix containing PCR primers (Primer and probes sequences of this assay are not disclosed) and fluorescently labeled TaqMan probes (Catalog number A44177, Thermo Fisher Scientific, Waltham, MA, USA) using the company’s protocol suggested master mix (Absolute Q^TM^ DNA digital PCR Master Mix (5X), Catalog number A52490, Thermo Fisher Scientific). The DNA template near limiting dilution (around 10 ng of genomic DNA, when possible) was loaded onto a MAP plate (Cat number A53301, Thermo Fisher Scientific), partitioned in the microwells and PCR amplified with a 10 minutes precycle at 96 °C followed by 40 cycles with a denaturing temperature of 96 °C for 5 s and a TM of 60 °C on a QuantStudio™ 3D machine. Probe-specific fluorescent signals were subsequently analyzed on the QuantaStudio Absolute Q Digital PCR Software (Thermo Fisher Scientific) and visualized in a two-dimensional scatterplot. In the analysis, the specific signal of the *IDH1* R132C mutation was detected as a FAM signal and the wildtype signal was detected as a VIC signal.

## Data Availability

All scMRD DNA and protein sequencing data is available through GEO accession number GSE271629.

## Code availability

Code script used to perform computational analysis and generate visualizations for both the scDNA and protein data were completed using the Mission Bio DNA + Protein analysis Jupyter notebook, available at https://missionbio.github.io/mosaic/notebooks/curated_notebooks/dna-protein-multisample.html.
